# Clinical application of a modified platelet desialylation test for mechanistic characterization of platelet transfusion refractoriness

**DOI:** 10.1111/trf.70246

**Published:** 2026-04-29

**Authors:** Karen Ziza, Thamy Silva, Mateus Domentino, Marina Conrado, João Oliveira, Elyse Moritz, Akemi Chiba, Vanderson Rocha, Alfredo Mendrone‐Junior, José Bordin, Langhi Dante, Carla Dinardo

**Affiliations:** ^1^ Fundação Pró‐Sangue Hemocentro de São Paulo São Paulo São Paulo Brazil; ^2^ Department of Hematology Universidade Federal de São Paulo (UNIFESP) São Paulo São Paulo Brazil; ^3^ Institute of Health Sciences Universidade Paulista São Paulo São Paulo Brazil; ^4^ Department of Hematology Faculdade de Medicina da Universidade de São Paulo (FMUSP) São Paulo São Paulo Brazil; ^5^ Churchill Hospital, NHS BT Oxford University Oxford UK

**Keywords:** desialylation, platelet refractoriness, platelet transfusion

## Abstract

**Background:**

Platelet transfusion refractoriness (PTR) is a major challenge in transfusion medicine and may result from both immune and non‐immune mechanisms. Although alloantibodies are well‐established contributors, Fc‐independent pathways such as platelet desialylation have emerged as alternative mechanisms of clearance.

**Study Design and Methods:**

In this prospective diagnostic study, 81 patients with suspected PTR were evaluated using an integrated approach combining the platelet immunofluorescence test (PIFT) to detect antibody‐mediated refractoriness and a modified platelet desialylation test (PDT) to assess Fc‐independent clearance. Demographic, clinical, and laboratory variables were analyzed using non‐parametric tests, including Mann–Whitney *U*, Kruskal–Wallis, and binomial tests.

**Results:**

The cohort exhibited severe thrombocytopenia and diverse clinical profiles. PIFT detected platelet‐bound antibodies in 63/81 cases (78%), while PDT identified desialylation in 41/81 patients (50%). PIFT positivity was similar between PDT‐positive (80.5%) and PDT‐negative (76.9%) groups, with no significant association (*χ* ^2^ = 0.15, *p* = .69). Although mean fluorescence intensity (MFI) varied across diagnostic categories, no significant differences were observed (*p* = .3095). Fever was the only clinical variable significantly associated with reduced desialylation (median MFI 401 vs. 1328.3; *p* = .0109), while splenomegaly, infection, antifungal use, and bleeding had no significant effects. PDT status was not significantly associated with the number of pooled platelet transfusions (*p* = .391) or apheresis procedures (*p* = .515), indicating that desialylation alone does not predict transfusion demand.

**Discussion:**

PTR occurs both independently and in parallel with antibody‐mediated pathways. The combined PIFT‐modified PDT approach improves mechanistic characterization and enhances diagnostic accuracy, in cases of platelet refractoriness of immune and non‐immune etiology.

**Abbreviations**
ABO: ABO blood group system
ALL: acute lymphoblastic leukemia
AML: acute myeloid leukemia
AMR: Ashwell–Morell Receptor
Anti‐HLA: anti‐human leukocyte antigen
Anti‐HPA: anti‐human platelet antigen
a.u.: arbitrary units
CCI: corrected count increment
DIC: disseminated intravascular coagulation
EDTA: ethylenediaminetetraacetic acid
ELISA: enzyme‐linked immunosorbent assay
Fc: fragment crystallizable region
FSC: forward scatter
HLA: human leukocyte antigen
HPA: human platelet antigen
HRP: horseradish peroxidase
IQR: interquartile range
MAIPA: monoclonal antibody immobilization of platelet antigens
MFI: mean fluorescence intensity
PBS: phosphate‐buffered saline
PDT: platelet desialylation test
PIFT: platelet immunofluorescence test
PTR: platelet transfusion refractoriness
RCA: Ricinus Communis Agglutinin
SD: standard deviation

## 1 INTRODUCTION

Platelet transfusion refractoriness (PTR) is defined as the repeated failure to obtain adequate post‐transfusion platelet count increments, typically assessed using the corrected count increment (CCI). PTR remains a major challenge in transfusion medicine because it increases bleeding risk, prolongs hospitalization, and negatively impacts treatment outcomes in hematologic and oncologic patients.^1^, ^2^, ^3^, ^4^, ^5^, ^6^

The causes of PTR are broadly categorized as non‐immune and immune. Non‐immune factors account for the majority of cases and include fever, active infection, disseminated intravascular coagulation (DIC), splenomegaly, and drug‐related effects. Immune‐mediated PTR primarily results from alloantibodies against human leukocyte antigen (HLA) class I molecules, while antibodies targeting human platelet antigens (HPA), ABO incompatibility, or CD36 deficiency contribute to a smaller proportion of cases. Anti‐HLA antibodies represent the predominant immune mechanism, with anti‐HPA or mixed specificities occurring less frequently.^3^, ^7^, ^8^

Several diagnostic tools are used to characterize alloimmunization and guide transfusion support. The monoclonal antibody immobilization of platelet antigens (MAIPA) assay remains the reference technique for identifying anti‐HPA antibodies. For anti‐HLA detection, current practice has shifted from complement‐dependent cytotoxicity assays to more sensitive solid‐phase methods, including Enzyme‐Linked Immunosorbent Assay (ELISA)‐based platforms and bead‐based Luminex technology. Flow cytometry‐based assays, such as the platelet immunofluorescence test (PIFT) or platelet crossmatch, provide rapid assessment of antibody–platelet interactions and are frequently used in specialized laboratories.^9^, ^10^, ^11^, ^12^

Notably, a subset of patients continues to exhibit poor platelet increments even when transfused with HLA‐matched or crossmatch‐compatible platelet units. This suggests the involvement of alternative mechanisms of platelet clearance that are independent of Fcγ receptor‐mediated splenic phagocytosis.^13^, ^14^

One mechanism of growing interest is platelet desialylation, in which neuraminidases remove terminal sialic acid residues from platelet glycoproteins, exposing β‐galactose and promoting hepatic clearance via the Ashwell–Morell receptor (AMR) clearance pathway.^15^, ^16^, ^17^ Platelet desialylation may occur in response to infections, platelet aging, high shear stress, storage lesions, hemostatic activation, or interactions with autoantibodies.^18^, ^19^, ^20^, ^21^, ^22^

Given these observations, evaluating platelet desialylation may provide complementary insights into the pathophysiology of both immune and non‐immune PTR, particularly in cases where conventional antibody testing does not fully explain transfusion outcomes.

The objective of this study was to investigate the role of platelet desialylation in PTR within a heterogeneous cohort of patients with suspected immune and non‐immune mechanisms, and to evaluate the clinical applicability of a modified platelet desialylation test (PDT) in the diagnostic assessment of patients undergoing repeated platelet transfusions.

## 2 METHODS

### 2.1 Study design and setting

This prospective, observational diagnostic study was conducted at the institutional transfusion service. Consecutive patients presenting clinical suspicion of PTR were systematically evaluated between March 2021 and June 2025. All participants provided written informed consent prior to enrollment. The study protocol was reviewed and approved by the institutional Blood Bank Ethics Committee, and all procedures were performed in accordance with relevant regulatory and ethical guidelines.

### 2.2 Patient selection

Patients were selected based on clinical evaluation and transfusion history, characterized by repeated platelet transfusions with inadequate post‐transfusion increments. To assess refractoriness including immune and non‐immune causes as well as potential hepatic clearance mechanisms, at least two consecutive post‐transfusion platelet increments were considered.

The CCI was used as a screening tool rather than a strict inclusion criterion. Accordingly, patients with CCI ≥5000 were retained when clinical and laboratory findings were consistent with an inadequate transfusion response. Importantly, inclusion was not restricted to CCI values, and patients with higher platelet counts were also included when clinically relevant, particularly considering their transfusion history and underlying disease.

Individuals with incomplete transfusion records, missing laboratory data, or insufficient follow‐up were excluded. Clinical, transfusion, and laboratory information was collected according to standardized institutional protocols to ensure consistency and data reliability.

All enrolled patients underwent a comprehensive evaluation to differentiate immune from non‐immune causes of PTR. Clinical factors known to impair platelet recovery, including active infection, fever, splenomegaly, disseminated intravascular coagulation, and exposure to medications affecting platelet kinetics, were systematically reviewed to identify potential non‐immune contributors.

### 2.3 Evaluation of immune and non‐immune mechanisms in platelet transfusion refractoriness

Immune‐mediated mechanisms were assessed using platelet crossmatching by the PIFT. Patients with positive results were classified as having immune‐mediated refractoriness, whereas those with negative results were considered not attributable to detectable immune mechanisms.

All patients underwent both PIFT and the modified PDT, allowing the simultaneous evaluation of antibody‐mediated platelet destruction and Fc‐independent mechanisms, such as platelet desialylation.

Patients with persistently poor platelet increments despite negative crossmatch results and transfusion with compatible units were classified as having refractoriness not attributable to detectable immune or non‐immune mechanisms. In these cases, the modified PDT was used to investigate platelet desialylation as a potential mechanism of platelet clearance.

### 2.4 Blood sample and preparation

Peripheral blood samples (4 mL) were collected from 81 patients into Vacutainers containing 7.5% K3 Ethylenediaminetetraacetic acid (EDTA), and plasma was separated by centrifugation at 1300 × *g* for 10 min. The CCI was assessed at least twice, 1 h after transfusion of ABO‐identical platelet concentrates, and used as a screening parameter to identify suboptimal platelet responses. Patients with low CCI values (<5000/μL) were further evaluated for immune‐mediated refractoriness.

### 2.5 Platelet refractoriness investigation

The PIFT was performed as a crossmatch between donor platelets and patient serum to detect antibodies against HLA class I and/or HPA antigens, using flow cytometry, following the methodology described by von dem Borne et al.^23^ and standardized within the routine workflow of our laboratory. For each patient, crossmatches were conducted with three to five platelet units from apheresis procedures. Platelets from these units were pooled platelet transfusions, and the pooled platelet transfusions samples were subsequently analyzed using the modified PDT.^24^ The modified PDT protocol was further modified, validated, and optimized to ensure applicability in both laboratory and clinical settings.

### 2.6 Modified platelet desialylation test protocol

For each assay, three apheresis platelet units collected within 24 h were pooled platelet transfusions. Platelets were washed twice with phosphate‐buffered saline (PBS) containing 1.0 × 10^−3^ (0.1%) EDTA (Sigma‐Aldrich, St. Louis, MO, USA) and centrifuged at 250 × g for 2 min. The final platelet concentration was adjusted to (1.5–2.5) × 10^11^/L using the Sysmex XN‐550 hematology analyzer. Fifty microliters of the resulting platelet suspension were added to each well of a microplate and centrifuged to promote adherence. For reagent validation, 2.0 × 10^2^ mmol/L β‐lactose (Sigma‐Aldrich) served as a negative control, while 5.0 × 10^−1^ U/mL *Clostridium perfringens* neuraminidase (NeuC) (Sigma‐Aldrich) served as a positive control. Patient plasma samples and pooled platelet transfusions plasma from healthy donors (five donors per pool) were added and gently mixed to minimize platelet activation, followed by incubation at 37°C for 30 min to allow antigen–antibody interactions.

Plates were washed three times with PBS/EDTA (1.0 × 10^−3^) and subsequently stained with Ricinus communis agglutinin (RCA) (Vector Laboratories, Burlingame, CA, USA) diluted in PBS/EDTA was added 5.0 × 10^−2^ L (50 μL) per well was added and incubated for 1 h protected from light. Following a final wash, platelets were resuspended in PBS/EDTA (1.0 × 10^−1^ L; 100 μL) for flow cytometry analysis using the DxFLEX instrument (Beckman Coulter, Brea, CA, USA), acquiring 1.0 × 10^4^ events (10.000 platelets) per sample. Mean fluorescence intensity (MFI) was recorded to quantify RCA binding. The PDT was developed based on the PIFT, with a detailed comparison between the two methods. Reference values were calculated using the statistical ratio (*R*), defined as *R* = median fluorescence of the sample/Median fluorescence of the normal reference control (healthy plasma pool). PDT results were classified as follows: *R* < 1.30, negative; 1.30 ≤ *R* ≤ 1.45, inconclusive; *R* > 1.45, positive. This ratio accounts for inter‐experimental and inter‐individual variability and was used to determine the presence or absence of platelet desialylation.

### 2.7 Clinical data collection

Clinical data were recorded using a standardized form and included age, sex, underlying diagnosis, fever within the last 24 h, infection, antifungal use, DIC, bleeding, and history of marrow transplantation.

### 2.8 Statistical analysis

Descriptive statistics were used to summarize all variables. Comparisons of MFI between clinical groups were performed using the Mann–Whitney *U* test, while differences across diagnostic categories were evaluated using the Kruskal–Wallis test. The association between PIFT and PDT categorical results was assessed using Pearson's chi‐square test.

The association between platelet count and MFI was evaluated using Spearman's rank correlation coefficient. To account for the potential influence of extreme values, correlation analyses were conducted both including all observations and after outlier removal based on the interquartile range (IQR) method.

Significance was defined as *p* < .05. All analyses were performed using RStudio (version 2024.04.2).

## 3 RESULTS

### 3.1 Demographic and clinical characteristics

The study population comprised 81 patients, with a predominance of women (65.4%) compared to men (34.6%). The overall median age was 50 years (range, 20–86), with men presenting a higher median age (57 years) than women (48 years). Regarding clinical characteristics, 45 patients (56%) had benign diseases and 36 (44%) had malignant conditions, with comparable sex distributions.

Among clinical manifestations, infection was the most frequent finding (32%), followed by bleeding (23%) and fever within the last 24 h (21%). Splenomegaly was documented in 11% of patients, and 21% were receiving antifungal therapy, predominantly women (71%). A total of nine patients (11%) had undergone marrow transplantation, occurring more frequently in men. Platelet counts were markedly reduced across the cohort, with a mean of 11 × 10^9^/L and a median of 8 × 10^9^/L, reflecting the severity of thrombocytopenia in this population (Table 1).

**TABLE 1:** Patient demographics and clinical profile.

Demographic characteristics			
Characteristics	Total	Women	Men
Sex	81 (100%)	53 (65%)	28 (35%)
Age, years: median	50 [20–86]	48 [26–86]	57 [20–74]
Underlying diagnoses			
Acute myeloid leukemia	18 (22.0%)	12 (67%)	6 (33%)
Acute lymphoblastic leukemia	16 (19.5%)	11 (69%)	5 (31%)
Aplastic anemia	13 (15.9%)	7 (54%)	6 (46%)
Myelodysplastic syndrome	9 (11.0%)	5 (56%)	4 (44%)
Evans syndrome	7 (8.5%)	5 (71%)	2 (29%)
Breast cancer	3 (3.7%)	3 (100%)	0
Multiple myeloma	3 (3.7%)	2 (67%)	1 (33%)
Chronic lymphocytic leukemia	2 (2.4%)	1 (50%)	1 (50%)
Non‐Hodgkin lymphoma	2 (2.4%)	1 (50%)	1 (50%)
Other diagnoses^a^	8 (9.8%)	6 (75%)	2 (25%)
Clinical characteristics			
Fever in the last 24 h	17 (21%)	10 (59%)	7 (41%)
Splenomegaly	9 (11%)	4 (44%)	5 (56%)
Infection	26 (32%)	17 (65%)	9 (35%)
Use of antifungal agents	17 (21%)	12 (71%)	5 (29%)
Bleeding	19 (23%)	13 (68%)	6 (32%)
Marrow transplantation	9 (11%)	3 (33%)	6 (67%)
Mean platelet count (SD)	11 × 10^9^/L	9 × 10^9^/L	11 × 10^9^/L
Median platelet count (IQR)	8 × 10^9^/L	7 × 10^9^/L	8 × 10^9^/L

Abbreviation: IQR, interquartile range. Standard Deviation (SD).

^a^ Chronic myeloid leukemia, myelofibrosis, antiphospholipid syndrome, Crohn's disease, Sjögren syndrome, lupus, endocarditis, and heart failure.

### 3.2 Laboratory investigation

**FIGURE 1:**
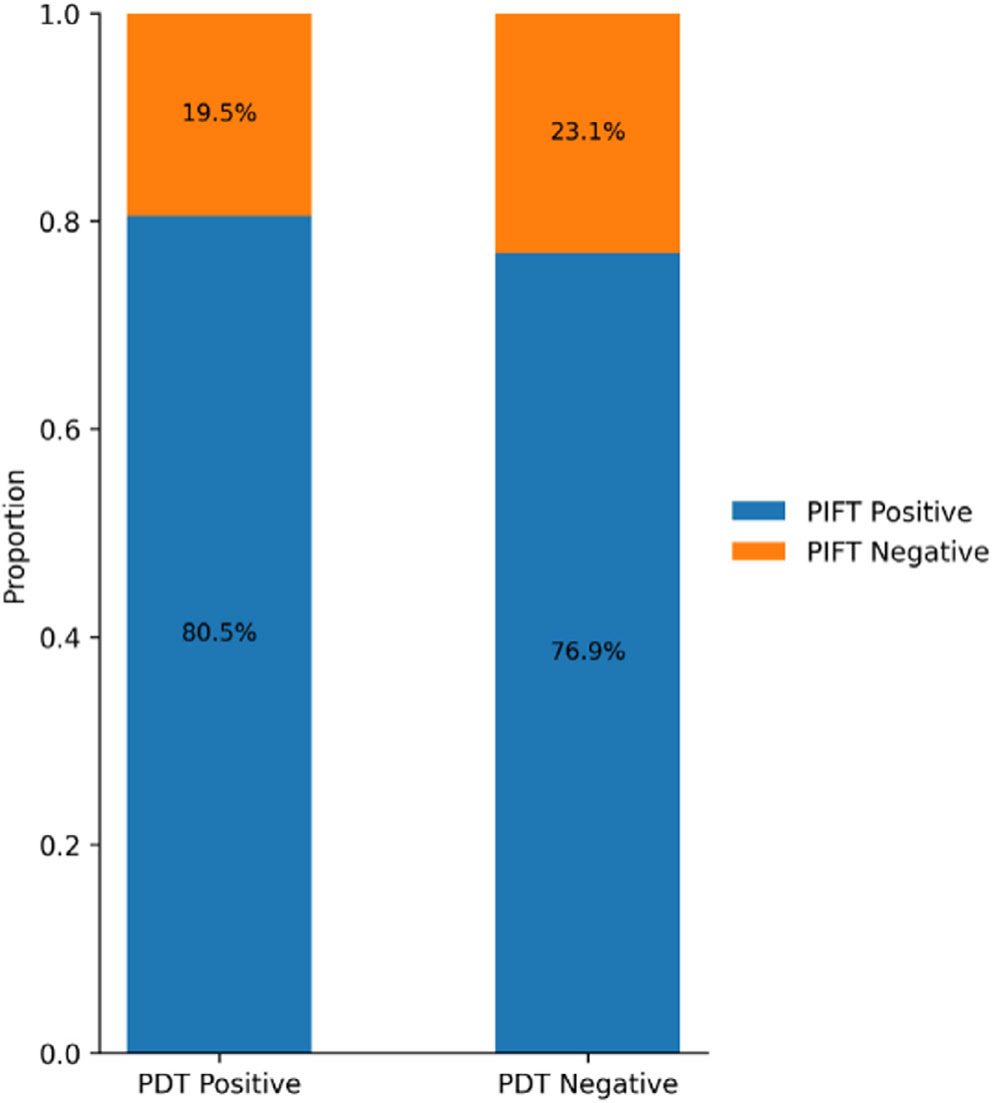
Distribution of platelet immunofluorescence test (PIFT) results according to platelet desialylation test (PDT) status. Stacked bar chart illustrating the proportion of PIFT‐positive and PIFT‐negative results within PDT‐positive and PDT‐negative groups. Among PDT‐positive samples, 80.5% were PIFT‐positive and 19.5% were PIFT‐negative, whereas among PDT‐negative samples, 76.9% were PIFT‐positive and 23.1% were PIFT‐negative. The distribution of PIFT results was similar across PDT groups, indicating no significant association between the tests (*χ* ^2^ = 0.15, *p* = .69, Pearson's chi‐square test).

Among the 81 samples evaluated, PIFT reactivity was detected in 63 cases (78%), while 18 samples (22%) tested negative. Using the modified PDT, 41 samples (50.0%) were classified as positive, 39 (47.6%) as negative, and 1 sample (1.2%) yielded an inconclusive result. Among the 41 PDT‐positive cases (Ratio >1.45), 33 (80.5%) were also PIFT‐positive, whereas only 8 (19.5%) were PIFT‐negative. Similarly, among the 39 PDT‐negative samples, 30 (76.9%) were PIFT‐positive and 9 (23.1%) were PIFT‐negative. Overall, the distribution of PIFT reactivity was comparable between PDT‐positive and PDT‐negative groups, with no significant association observed between the two assays (Pearson's chi‐square test, *χ* ^2^ = 0.15, *p* = .69), suggesting that platelet desialylation and PIFT reactivity may represent partially independent mechanisms in this cohort (Figure 1, Table A1).

Platelet counts, MFI values, and ratio measurements for all patients are presented in (Table A2). Median values and IQRs were calculated for individuals who tested positive by the modified PDT and subsequently compared across clinical diagnoses and manifestations, as detailed in (Table A3).

**FIGURE 2:**
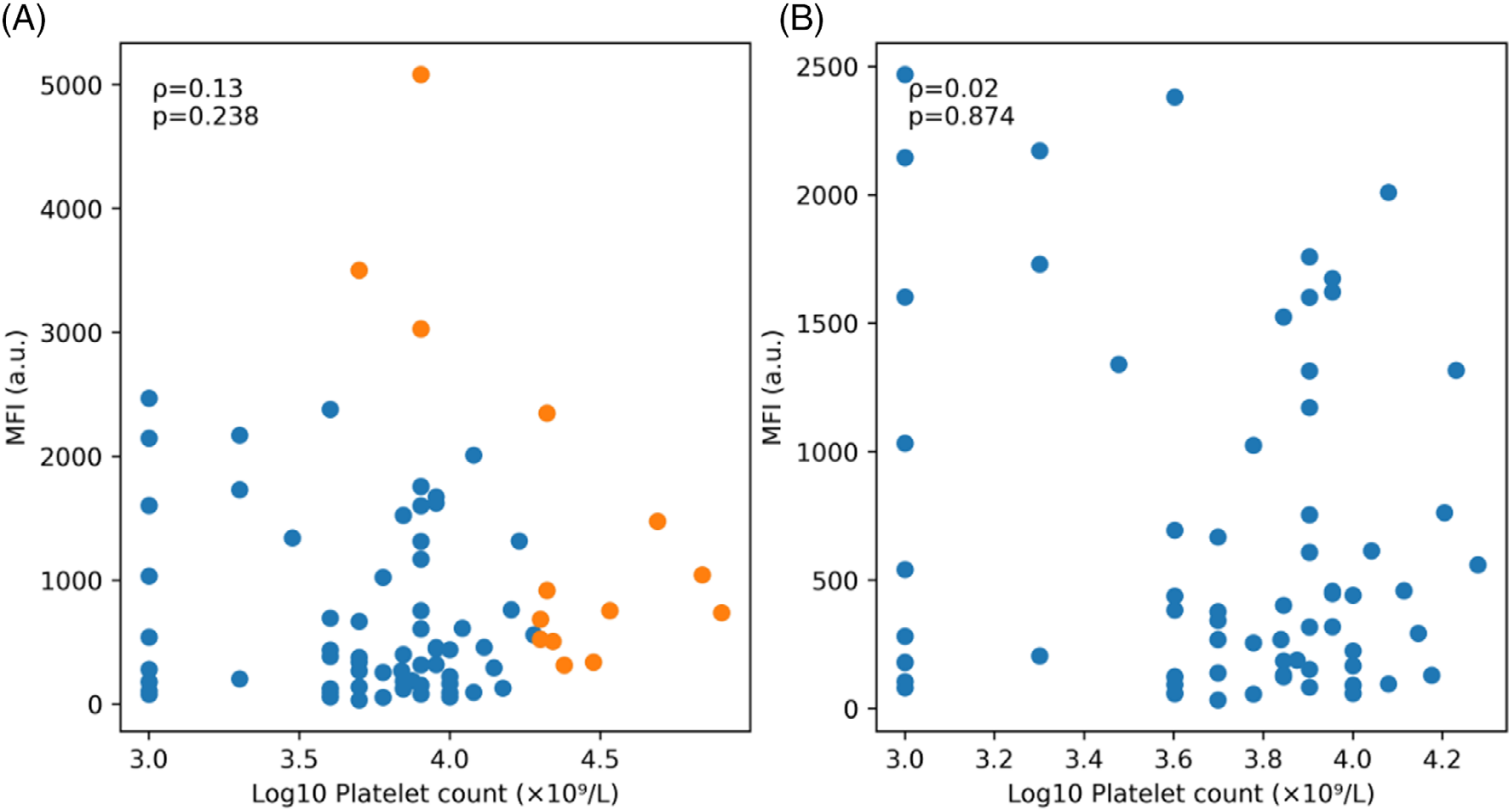
Relationship between platelet count and mean fluorescence intensity (MFI) assessed by Ricinus communis agglutinin (RCA)‐I binding. Platelet counts were log10‐transformed to account for skewed distribution. (A) Analysis including all observations, with outliers highlighted (orange) and defined according to Tukey's 1.5 × IQR rule. (B) Analysis after exclusion of outliers. No significant correlation was observed between platelet count and MFI in either analysis (Spearman's *ρ* = 0.13, *p* = .238 for all data; *ρ* = 0.02, *p* = .874 after outlier removal).

Platelet count was compared with MFI, and no significant correlation was observed between these variables. Using Spearman's rank correlation, the analysis including all samples demonstrated a weak and non‐significant association (*ρ* = 0.13, *p* = .238). After outlier removal based on the IQR method, the results remained consistent, with no significant correlation detected (*ρ* = 0.02, *p* = .874). These findings indicate that platelet desialylation levels, as measured by MFI, are not directly associated with absolute platelet counts in the studied population (Figure 2A,B).

### 3.3 Diagnostic and clinical predictors of platelet desialylation

Analysis of mean MFI values with corresponding 95% confidence intervals among PDT‐positive patients demonstrated considerable variability across diagnostic categories. The highest desialylation levels were observed in Crohn's disease, Sjögren syndrome, myelodysplastic syndrome, and antiphospholipid syndrome, all showing markedly elevated MFI values accompanied by wide confidence intervals, consistent with heterogeneous biological responses within these groups. Acute Myeloid Leukemia (AML) and Acute Lymphoblastic Leukemia (ALL) exhibited intermediate MFI levels with moderately narrow intervals, whereas chronic lymphocytic leukemia, chronic myeloid leukemia, and multiple myeloma displayed the lowest mean intensities, suggesting minimal involvement of desialylation pathways in these hematologic malignancies.

**FIGURE 3:**
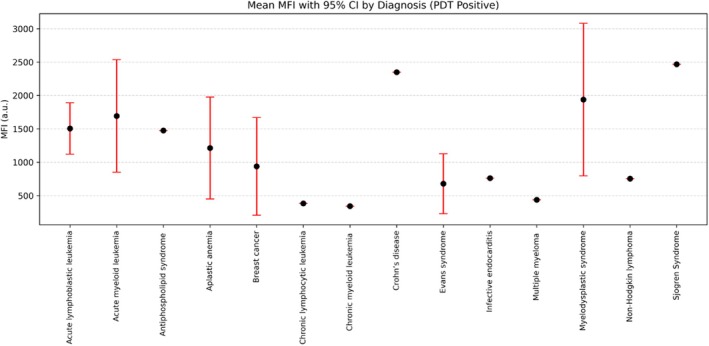
PDT‐Positive Patients by Diagnosis. Each point represents the mean MFI (a.u.) for patients within a diagnostic category, with vertical bars indicating the corresponding 95% confidence interval. Substantial variability in desialylation levels was observed across clinical groups, with higher MFI values particularly notable in myelodysplastic syndrome, Crohn’s disease, and Sjögren syndrome, whereas lower MFI values appeared in chronic leukemias and multiple myeloma. These findings highlight the heterogeneity of platelet desialylation across distinct hematologic and immune‐mediated disorders. CI, confidence interval.

Despite these visual trends, no significant differences were detected in MFI distribution across diagnostic groups (Kruskal–Wallis: *H* = 14.96, *p* = .3095), indicating that the observed variation is more likely attributable to sample size constraints and intrinsic clinical heterogeneity rather than true diagnosis‐specific effects (Figure 3).

When clinical manifestations were compared with PDT fluorescence intensity, fever in the preceding 24 h was the only variable associated with a significant reduction in MFI (median 401 vs. 1328.3; *p* = .0109). Although patients with splenomegaly showed lower median MFI values compared with those without (736.7 vs. 1328.3), this difference did not reach significance (*p* = .0904). No significant associations were observed for infection (*p* = .2233), antifungal use (*p* = .7684), or bleeding (*p* = .5000) (Table 2).

**TABLE 2:** Clinical predictors among patients with a positive modified platelet desialylation test.

Clinical variable	*n* (yes)	Median MFI (yes)	*n* (No)	Median MFI (No)	*p*‐Value^a^	Interpretation
Fever in the last 24 h	7	401	34	1328.3	.0109	Significant—fever associated with lower MFI
Splenomegaly	5	736.7	36	1328.3	.0904	Trend, not significant
Infection	12	893.3	29	1339.9	.2233	Not significant
Use of antifungal agents	7	1032.3	34	1244	.7684	Not significant
Bleeding	8	1028.3	33	1316.7	.5	Not significant

Abbreviation: MFI, mean fluorescence intensity.

^a^ Mann–Whitney *U*.

**FIGURE 4:**
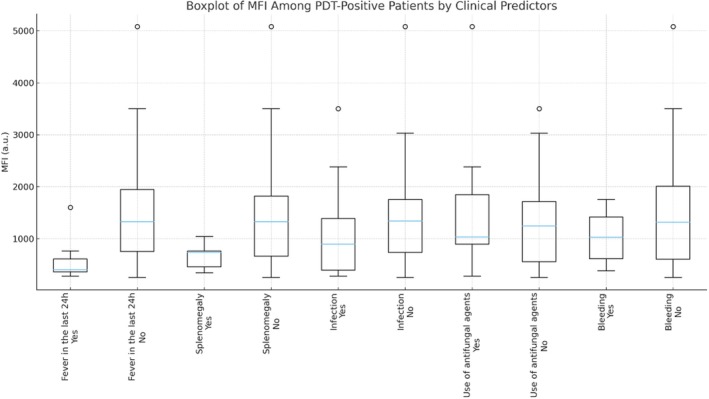
Distribution of MFI values among PDT‐positive patients stratified by clinical predictors. Boxplots illustrate the variability of MFI values (a.u.) in PDT‐positive patients, stratified according to the presence or absence of fever within the last 24 hours, splenomegaly, infection, use of antifungal agents, and bleeding. Central lines represent medians; boxes indicate interquartile ranges; whiskers denote data dispersion; and circles represent outliers. Comparisons between groups (yes vs. no) were performed using the Mann–Whitney U test. No statistically significant differences in MFI distributions were observed across clinical subgroups (all p > 0.05), indicating that desialylation levels among PDT‐positive patients are heterogeneous but not primarily driven by these clinical variables. Distribution of Transfusion Counts by platelet desialylation test (PDT) classification. (A) Distribution of the number of pooled platelet transfusions platelet transfusions among patients with inconclusive, negative, and positive PDT results. (B) Distribution of the number of apheresis platelet transfusions across the same PDT categories. Boxplots display medians, interquartile ranges, whiskers indicating data dispersion, and outliers. Patients with positive PDT results tended to receive a higher number of transfusions, both pooled platelet transfusions and apheresis, compared with PDT‐negative individuals, whereas inconclusive results showed limited variability due to the small sample size. These findings suggest a potential association between platelet desialylation and increased transfusion requirements. MFI, mean fluorescence intensity.

These findings were visually reinforced in the boxplot analysis (Figure 4), in which fever‐positive patients demonstrated a consistently lower and more compressed MFI distribution, whereas all other clinical predictors displayed substantial overlap between categories and wide variability. Statistical comparisons between groups (yes vs. no) were performed using the Mann–Whitney *U* test, confirming that fever was the only clinical variable significantly associated with lower MFI values (*p* < .05), while no significant differences were observed for the remaining predictors. Collectively, these results indicate that fever is the only clinical manifestation meaningfully associated with reduced platelet desialylation among PDT‐positive patients.

### 3.4 Platelet transfusion requirements according to PDT status

The evaluation of total platelet transfusion requirements, considering both pooled platelet transfusions and platelets transfusions derived from apheresis procedures, revealed heterogeneous patterns across the different PDT result groups. For both transfusion types, patients with a positive PDT exhibited greater variability and wider dispersion in transfusion counts, including multiple outliers particularly evident in apheresis transfusions, in which some individuals exceeded 100 and even 300 units.

**FIGURE 5:**
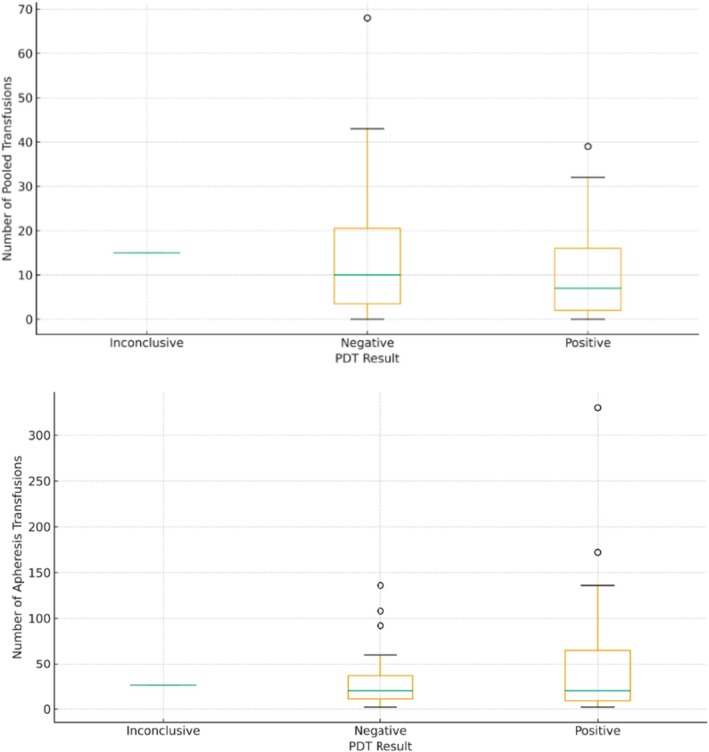
Distribution of Transfusion Counts by PDT Classification. (A) Distribution of the number of pooled platelet transfusions among patients with inconclusive, negative, and positive PDT results. (B) Distribution of the number of apheresis platelet transfusions across the same PDT categories. Boxplots display medians, interquartile ranges, whiskers indicating data dispersion, and outliers. Patients with positive PDT results tended to receive a higher number of transfusions both pooled platelet transfusions and apheresis compared with PDT‐negative individuals, whereas inconclusive results showed limited variability due to the small sample size. These findings suggest a potential association between platelet desialylation and increased transfusion requirements.

In contrast, PDT‐negative patients showed more compact distributions, concentrated within lower transfusion ranges, whereas the inconclusive group displayed intermediate and relatively stable values, although limited by its small sample size. Despite these visual differences observed in the boxplots, none of the comparisons reached significance for either pooled platelet transfusions platelets (*p* = .391) or apheresis platelet units (*p* = .515) (Figure 5A,B, Table A4).

These findings indicate that, although patients with a positive PDT may demonstrate greater transfusion variability, the PDT result alone was not significantly associated with the number of platelet transfusions required.

## 4 DISCUSSION

This study provides an integrated assessment of Fc‐dependent and Fc‐independent mechanisms of platelet clearance by combining the PIFT with a modified PDT. The heterogeneous clinical profile of the cohort, including immune, inflammatory, neoplastic, infectious, and post‐transplant conditions, reflects the multifactorial nature of thrombocytopenia and platelet refractoriness (PTR) in real‐world settings.

The high rate of PIFT reactivity (78%) reinforces the central role of alloimmune and autoimmune mechanisms in platelet destruction, consistent with previous studies. Notably, the substantial overlap between PIFT and modified PDT positivity suggests that platelet desialylation frequently coexists with antibody‐mediated platelet engagement.

However, the identification of PDT‐positive cases among PIFT‐negative patients highlights the presence of Fc‐independent pathways, supporting the concept that desialylation‐mediated clearance represents a distinct mechanism not captured by conventional serological assays. This dual‐mechanism model is consistent with emerging evidence demonstrating that platelet desialylation contributes to thrombocytopenia across both immune and non‐immune contexts and may occur independently of Fcγ receptor–mediated phagocytosis.^25^, ^26^, ^27^

Importantly, the relationship between platelet desialylation and transfusion response was further explored through correlation analysis between MFI and platelet count. The absence of a significant correlation between platelet count and MFI suggests that platelet desialylation is not directly dependent on circulating platelet levels. This finding supports the concept that desialylation represents an independent pathway of platelet clearance rather than a secondary consequence of thrombocytopenia severity. Notably, this lack of association persisted even after outlier removal, indicating that the observed results were not driven by extreme values and reinforcing the robustness of this finding.

In contrast to immunologic refractoriness, which often prevents achieving minimally safe platelet thresholds, non‐immune factors such as fever and systemic inflammation may influence platelet kinetics without completely abolishing transfusion response.

Variation in desialylation intensity across diagnostic categories suggests potential disease‐specific biological influences, with higher MFI values observed in immune‐inflammatory disorders and lower levels in marrow failure or hematologic malignancies. Although these trends did not reach significance, likely due to sample size and clinical heterogeneity, they highlight the need for larger mechanistic studies evaluating how inflammation, cytokine signaling, and enzymatic neuraminidase activity modulate platelet glycobiology in vivo.

Among clinical manifestations, fever emerged as the only factor associated with reduced desialylation activity. This inverse association may reflect dynamic alterations in platelet glycan profiles during acute inflammatory stress or accelerated removal of desialylated platelets in febrile states.^28^, ^29^ Other clinical variables such as splenomegaly, infection, antifungal exposure, and bleeding showed no consistent effect, emphasizing that desialylation is not uniformly modulated by all inflammatory or disease‐related stimuli.

The potential influence of pharmacological factors was also considered. Although antifungal agents were initially emphasized due to their frequency of use in this cohort, other medications are known to induce drug‐dependent platelet‐reactive antibodies. The lack of comprehensive medication data limited further analysis; however, this aspect underscores the complex interplay between immune and non‐immune mechanisms in PTR.

The absence of a significant association between PDT status and platelet transfusion burden further indicates that desialylation alone does not determine transfusion requirements. Although PDT‐positive patients displayed broader variability and extreme transfusion needs, these findings likely reflect underlying disease severity and transfusion practices rather than direct consequences of platelet desialylation. Overall, the results support the use of PDT as a complementary immunopathologic tool rather than a standalone predictor of transfusion demand.

Additionally, ABO blood group may influence lectin binding and, consequently, PDT results, given the role of glycosylation patterns in platelet surface biology. Although no subgroup analysis was performed due to sample size constraints, this factor represents an important biological variable that warrants further investigation in future studies.

Methodologically, although platelet size can influence lectin binding, standardized Forward Scatter (FSC)/Side Scatter (SSC) gating strategies were applied to ensure analysis of a homogeneous platelet population, minimizing potential bias. Nevertheless, alternative normalization approaches, such as FSC‐based correction or adjustment by mean platelet volume, may further refine the accuracy of desialylation measurements.

Taken together, the combined application of PIFT and PDT provides a more comprehensive characterization of platelet clearance mechanisms. While antibody testing remains fundamental for detecting alloimmune and autoimmune responses, PDT offers mechanistic insight into Fc‐independent pathways, particularly in patients with persistent thrombocytopenia despite negative antibody screens. The recognition that platelet desialylation is a modifiable pathway carries therapeutic relevance; neuraminidase inhibitors such as oseltamivir have shown potential to attenuate desialylation‐mediated clearance and improve platelet counts.^30^, ^31^, ^32^, ^33^ Patients who are PIFT‐negative but PDT‐positive may be particularly suitable candidates for such targeted interventions.

This study is limited by its small sample size and cohort heterogeneity, which precluded robust subgroup analyses. CCI was used as a screening tool and may not reflect clinically meaningful transfusion outcomes. Incomplete data on concomitant medications limited assessment of drug‐dependent platelet antibodies. MFI values were not normalized for platelet size, and the cross‐sectional design precludes causal inference.

In summary, this study demonstrates that platelet desialylation is a frequent and mechanistically important contributor to thrombocytopenia and platelet refractoriness. Integrating modified PDT into routine immunohematologic evaluation enhances diagnostic resolution and may support individualized therapeutic strategies, particularly in complex or refractory cases.

## 5 CONCLUSIONS

In summary, the modified PDT identified platelet desialylation as a frequent and clinically relevant mechanism of clearance, complementing antibody detection by PIFT. The combined use of both assays improved mechanistic classification of thrombocytopenia, revealing Fc‐independent pathways even in PIFT‐negative cases. Although desialylation intensity varied across diagnoses, fever was the only clinical factor associated with reduced MFI. These findings support incorporating the modified PDT into routine immunohematologic evaluation to refine diagnosis and guide mechanism‐based management of platelet refractoriness.

## CONFLICT OF INTEREST STATEMENT

The authors have disclosed no conflicts of interest.

## Data Availability

The data that support the findings of this study are available from the corresponding author upon reasonable request.

## APPENDIX A

**TABLE A1:** Distribution of platelet immunofluorescence test (PIFT) and modified platelet desialylation test (PDT) test results in the study population.

Laboratory variable	Category	*n*	Proportion (%)
PIFT	Positive	63	78%
PIFT	Negative	18	22%
Modified PDT test	Positive	41	50%
Modified PDT test	Negative	39	48%
Modified PDT test	Inconclusive	1	1.2%
PIFT among PDT‐modified samples	Positive	33	80.49%
PIFT among PDT‐modified samples	Negative	8	19.51%
PIFT among PDT‐modified samples	95% CI (Positive)	‐	0.6599–0.8977

Abbreviation: CI, confidence interval.

**TABLE A2:** Clinical and immunohematologic characteristics with platelet immunofluorescence test (PIFT) and platelet desialylation test (PDT) results.

Sample	Age	Sex	Diagnostic	Blood group	PIFT	PDT	Platelet count (SD)	MFI (a.u.) PDT	Ratio
1	67	Female	Chronic lymphocytic leukemia	A+	Positive	Negative	5000	667.2	0.74
2	20	Male	Aplastic anemia	O+	Positive	Negative	4000	123.5	0.98
3	20	Male	Aplastic anemia	O+	Negative	Positive	1000	280.3	2.24
4	46	Female	Aplastic anemia	O+	Positive	Positive	5000	377.5	3.01
5	60	Female	Aplastic anemia	O+	Positive	Positive	5000	3501	2.12
6	66	Female	Acute lymphoblastic leukemia	O+	Positive	Positive	4000	2380.4	2.65
7	55	Female	Acute myeloid leukemia	A+	Negative	Positive	1000	2145.1	2.39
8	62	Male	Acute myeloid leukemia	A+	Negative	Positive	21,000	918.8	1.79
9	61	Male	Acute myeloid leukemia	AB+	Positive	Positive	8000	754.2	1.47
10	64	Male	Acute myeloid leukemia	O+	Positive	Positive	7000	401	3.2
11	78	Female	Acute myeloid leukemia	O+	Positive	Negative	14,000	292.7	0.82
12	32	Female	Non‐Hodgkin lymphoma	A+	Positive	Negative	6000	55.5	0.44
13	57	Male	Acute myeloid leukemia	O‐	Positive	Negative	4000	694.1	0.77
14	65	Female	Multiple myeloma	A+	Negative	Negative	7500	188.4	0.75
15	33	Female	Multiple myeloma	O+	Positive	Negative	19,000	558.9	0.62
16	69	Male	Myelodysplastic syndrome	O+	Negative	Negative	10,000	166.4	0.68
17	46	Female	Crohn's disease	O+	Positive	Positive	21,000	2349.1	6.61
18	41	Female	Aplastic anemia	B‐	Positive	Positive	1000	1601.9	1.78
19	43	Male	Acute lymphoblastic leukemia	A+	Negative	Negative	11,000	613.2	0.68
20	53	Male	Acute lymphoblastic leukemia	A+	Positive	Negative	8000	316.4	1.26
21	47	Female	Acute lymphoblastic leukemia	O+	Positive	Negative	9000	447	0.5
22	48	Female	Acute lymphoblastic leukemia	B+	Positive	Positive	9000	1621.8	1.81
23	47	Female	Acute lymphoblastic leukemia	O+	Positive	Negative	7000	122.9	0.98
24	44	Female	Acute lymphoblastic leukemia	O+	Positive	Negative	9000	456	0.51
25	71	Female	Acute lymphoblastic leukemia	O+	Positive	Positive	6000	1024.3	1.75
26	46	Male	Chronic myeloid leukemia	A+	Positive	Positive	5000	341.8	2.73
27	50	Male	Evans syndrome	O+	Positive	Positive	80,000	736.7	2.07
28	21	Male	Myelodysplastic syndrome	O+	Positive	Negative	4000	57.5	0.23
29	40	Female	Acute myeloid leukemia	O+	Positive	Positive	8000	5080.7	3.08
30	67	Male	Aplastic anemia	O+	Negative	Negative	22,000	507.6	0.56
31	60	Male	Acute myeloid leukemia	B+	Negative	Positive	12,000	2009.5	2.24
32	45	Female	Acute myeloid leukemia	O+	Positive	Negative	8000	152.6	1.22
33	61	Male	Non‐Hodgkin lymphoma	AB+	Negative	Positive	34,000	754.2	1.47
34	23	Male	Acute myeloid leukemia	A‐	Positive	Positive	8000	1600.5	1.78
35	65	Male	Aplastic anemia	O+	Positive	Positive	2000	1729.6	1.92
36	67	Male	Multiple myeloma	O‐	Positive	Positive	4000	437.5	3.49
37	48	Female	Antiphospholipid syndrome	O+	Positive	Positive	49,000	1476.1	4.15
38	37	Female	Acute myeloid leukemia	O+	Positive	Negative	1000	104.6	0.83
39	40	Female	Aplastic anemia	A+	Positive	Positive	5000	267.7	2.13
40	50	Female	Congestive heart failure	O+	Positive	Negative	4000	92.4	0.74
41	50	Female	Evans syndrome	O+	Positive	Negative	4000	123.8	0.99
42	32	Female	Acute myeloid leukemia	O+	Positive	Negative	7000	185.1	0.74
43	45	Female	Breast cancer	O+	Positive	Positive	9000	1673.3	1.86
44	47	Female	Infective endocarditis	O+	Positive	Positive	16,000	762.3	1.49
45	61	Male	Acute lymphoblastic leukemia	A‐	Positive	Positive	8000	1171.3	1.5
46	69	Male	Myelodysplastic syndrome	O+	Negative	Negative	24,000	314.7	0.88
47	38	Male	Acute lymphoblastic leukemia	A+	Positive	Negative	5000	137.8	0.55
48	59	Female	Acute lymphoblastic leukemia	AB+	Positive	Positive	7000	1523.8	1.7
49	60	Female	Evans syndrome	O+	Positive	Positive	69,000	1042.8	2.93
50	63	Female	Aplastic anemia	O+	Negative	Negative	9000	318.3	0.35
51	30	Female	Acute myeloid leukemia	A+	Positive	Positive	8000	1314.5	1.83
52	42	Female	Acute myeloid leukemia	O+	Positive	Negative	10,000	90.5	0.36
53	73	Female	Myelodysplastic syndrome	O‐	Positive	Negative	1000	79.6	0.32
54	56	Female	Myelodysplastic syndrome	B+	Positive	Negative	4000	59.1	0.24
55	26	Female	Acute lymphoblastic leukemia	A+	Positive	Negative	10,000	440.4	0.49
56	46	Female	Aplastic anemia	A+	Positive	Positive	3000	1339.9	1.49
57	48	Female	Acute lymphoblastic leukemia	B+	Negative	Negative	20,000	523.3	0.58
58	44	Female	Aplastic anemia	A+	Positive	Negative	2000	204.7	0.82
59	31	Female	Evans syndrome	B+	Positive	Negative	8000	82.2	0.66
60	32	Female	Evans syndrome	B+	Positive	Positive	6000	255.6	2.04
61	75	Female	Sjogren Syndrome	A+	Negative	Positive	1000	2467.9	2.75
62	57	Male	Acute lymphoblastic leukemia	A+	Positive	Positive	17,000	1316.7	1.47
63	59	Female	Acute myeloid leukemia	A+	Positive	Positive	2000	2171.2	2.42
64	28	Female	Breast cancer	O+	Positive	Negative	15,000	129.3	1.03
65	28	Female	Breast cancer	O+	Positive	Positive	20,000	683.7	5.45
66	74	Male	Breast cancer	O+	Negative	Positive	13,000	458.5	1.83
67	20	Male	Aplastic anemia	A+	Negative	Negative	1000	180.4	0.72
68	47	Male	Chronic lymphocytic leukemia	O+	Positive	Positive	4000	383.1	3.06
69	45	Female	Acute myeloid leukemia	O+	Positive	Negative	5000	32.2	0.13
70	51	Female	Systemic lupus erythematosus	O+	Positive	Negative	10,000	223.5	0.89
71	60	Female	Evans syndrome	O+	Positive	Negative	6900	267.8	1.07
72	86	Female	Myelodysplastic syndrome	A‐	Positive	Positive	8000	1757.9	1.96
73	71	Male	Myelodysplastic syndrome	A+	Positive	Negative	7000	129.9	1.04
74	59	Female	Myelodysplastic syndrome	O+	Positive	Positive	1000	1032.3	2.9
75	56	Female	Acute lymphoblastic leukemia	A+	Positive	Negative	10,000	58.1	0.23
76	41	Female	Acute myeloid leukemia	O+	Negative	Inconclusive	1000	178.6	1.42
77	60	Female	Acute lymphoblastic leukemia	A‐	Positive	Negative	12,000	95	0.76
78	26	Male	Evans syndrome	B+	Negative	Negative	30,000	338.8	0.38
79	26	Male	Aplastic anemia	O‐	Negative	Positive	8000	607.7	1.48
80	60	Female	Myelodysplastic syndrome	A+	Positive	Positive	8000	3027.7	1.83
81	65	Female	Acute myeloid leukemia	A+	Positive	Positive	1000	540.1	1.46
Mean	50.14						10,610	804.07	1.55
Median	50						8000	447.0	1.46
Standard deviation							13,020	905.86	1.15

Abbreviation: MFI, mean fluorescence intensity.

**TABLE A3:** Median and interquartile range of mean fluorescence intensity (MFI) and ratio values across diagnoses in platelet desialylation test‐positive patients.

Diagnostic	*n*	MFI_median	MFI_Q1	MFI_Q3	Ratio_median	Ratio_Q1	Ratio_Q3
Acute lymphoblastic leukemia	6	1420.25	1207.65	1597.3	1.725	1.55	1.795
Acute myeloid leukemia	10	1457.5	795.35	2111.2	2.035	1.783	2.413
Antiphospholipid syndrome	1	1476.1	1476.1	1476.1	4.15	4.15	4.15
Aplastic anemia	8	973.8	353.2	1633.8	2.02	1.708	2.158
Breast cancer	3	683.7	571.1	1178.5	1.86	1.845	3.655
Chronic lymphocytic leukemia	1	383.1	383.1	383.1	3.06	3.06	3.06
Chronic myeloid leukemia	1	341.8	341.8	341.8	2.73	2.73	2.73
Crohn's disease	1	2349.1	2349.1	2349.1	6.61	6.61	6.61
Evans syndrome	3	736.7	496.15	889.75	2.07	2.06	2.5
Infective endocarditis	1	762.3	762.3	762.3	1.49	1.49	1.49
Multiple myeloma	1	437.5	437.5	437.5	3.49	3.49	3.49
Myelodysplastic syndrome	3	1757.9	1395.1	2392.8	1.96	1.895	2.43
Non‐Hodgkin lymphoma	1	754.2	754.2	754.2	1.47	1,47	1.47
Sjogren syndrome	1	2467.9	2467.9	2467.9	2.75	2,75	2.75

**TABLE A4:** Platelet transfusion counts in apheresis procedures and pooled platelet transfusions platelet products by platelet desialylation test (PDT) group.

Transfusion type	PDT group	Median (IQR)	^a^ *p*‐Value
Apheresis procedures	Positive	20 (9–65)	.515
Apheresis procedures	Negative	20 (11–36.5)	
Pooled platelet transfusions	Positive	7 (2–16)	.391
Pooled platelet transfusions	Negative	10 (3.5–20.5)	

Abbreviation: IQR, interquartile range.

^a^ Mann–Whitney *U*.
